# League of Mercy

**Published:** 1906-04-07

**Authors:** 


### LEAGUE OF MERCY.

On Saturday, March 31, Lady Newnes invited a number of the residents of Putney to Wildcroft, to have the honour of meeting H.R.H. Princess Alexander of Teck, Lady President of the Kingston-Surbiton (with Wandsworth-Putney) District of the League of Mercy, and to hear Sir Henry Burdett explain the objects of the League. H.R.H., who presided at the meeting, called first upon Sir Henry Burdett, who began by speaking some very plain words about the slackness of Putney in the matter of hospital accommodation, pointing out that in Putney and Wandsworth there was a population of 230,000 and not a single hospital bed, while if Battersea was included there was a population of 430,000 with only a provision of 30 hospital beds. Passing from that topic to the development of the League of Mercy, Sir Henry showed how, within the few years that the League had been in existence, it has added about £60,000 to King Edward's Hospital Fund. The division in which Putney is included did not at first contribute largely to the funds of the League, but since Princess Alexander of Teck had become President the contributions had reached the amount of £500 a year. The growth of the League was evidenced by the fact that within the last five years there had been secured in London alone about half a million associates. Sir Henry emphasised the point, which is a cardinal characteristic of the work of the League, that all classes must be invited to join in its work. He suggested the appointment of captains of each street, who should make themselves responsible for the visiting of each inhabitant, to ask them to join as members, or at least as associates, and bade those who took up the work welcome, as part of their personal service, the snubs and refusals which they might meet in the course of their visits and requests for help. He pointed out how much could be done by the co-operation of the humbler classes of society, and instanced a rag and bone seller, who brought in 27s. as her contribution to the funds of the League last year. This poor woman always carries her card of membership tied round her neck, feeling sure that if, in her precarious life, she meets with an accident or falls ill, and has to be taken to a hospital, the possession of it will appeal to the doctors wherever she may be taken. The same note was struck by Mrs. Moreing, Lady President of the Esher Division of the League, who gave those present very practical advice as to the best methods of securing members. She mentioned charwomen as particularly large-hearted creatures, who were generally willing to give and collect all they could for the benefit of the sick poor. Mrs. Moreing's experience was that when she timidly asked for a shilling, she not only got it without a grumble, but often received the reproach, "Why didn't you ask me before?" She advised members not to be afraid of the word "overlapping"—implying the capture of a contributor by another member. That only meant that another member must be secured to keep up the balance. In fact, "overlapping" meant competition, which is the life of charity, as well as of other things. She recommended that vice-presidents should make each member sign his or her name in the book provided, and that associates should be compelled to accept the receipt for their contribution, as this made them feel that they were not casual donors, but belonged to a permanent organisation, and thus helped to make them permanent contributors. After the usual vote of thanks, and refreshments provided by the hospitality of Lady Newnes, the meeting terminated, having aroused an interest in the work of the League which should surely develop into large and practical support of its beneficent work.

